# Thermally Induced Morphological and Structural Transformations on Eu^2+^/Eu^3+^-Coactivated Calcium Silicate Nanophosphors

**DOI:** 10.1021/acsaom.3c00456

**Published:** 2024-02-27

**Authors:** Hyun-Joo Woo, Kay Hadrick, Taeho Kim

**Affiliations:** Department of Biomedical Engineering, Institute for Quantitative Health Science and Engineering, Michigan State University, East Lansing, Michigan 48824, United States

**Keywords:** Ca_2_SiO_4_, silicate, hydrothermal, Eu^2+^/Eu^3+^ coactivated, nanophosphors

## Abstract

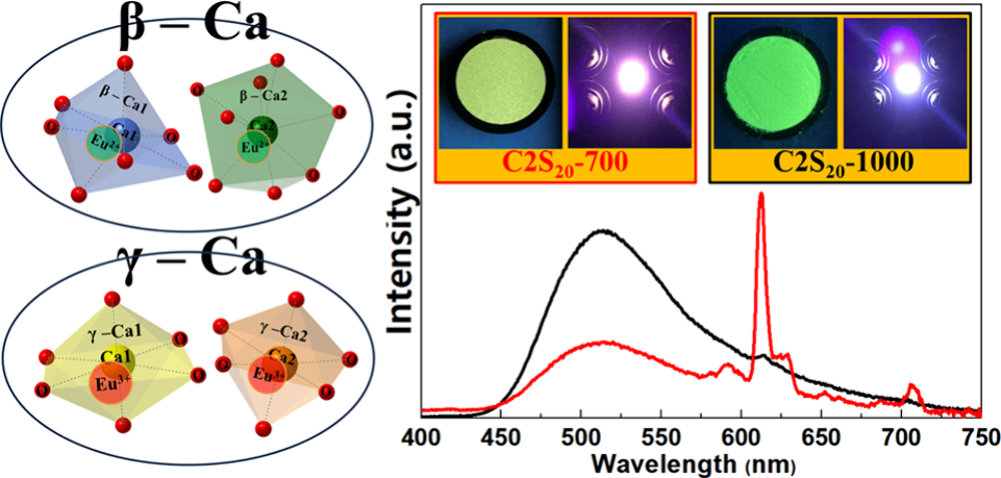

This study presents an approach for synthesizing Eu^2+^/Eu^3+^-coactivated Ca_2_SiO_4_ nanophosphors, by adjusting the ratio of both activators within a singular host material. Utilizing a hydrothermal method complemented by a postreduction sintering process, we fabricated a series of phosphors characterized by uniform 30–50 nm spherical nanoparticles. These engineered phosphors manifest multichannel luminescence properties and exhibit simultaneous blue and red emission from Eu^2+^ and Eu^3+^, respectively. Meticulous control of the 5% H_2_–95% N_2_ reduction temperature allowed for precise tuning of the Eu^2+^ and Eu^3+^ ions within the host lattice, which enabled the strategic adjustment of their luminescent outputs. Utilizing X-ray photoelectron spectroscopy (XPS), we could discern subtle alterations in the europium oxidation state. By using a transmission electron microscope (TEM) and an X-ray diffractometer (XRD), we found that the subsequent changes by reductive sintering to particle size, morphology, and mixed crystal structures influenced the materials’ luminescent characteristics. Our findings herald a significant advancement in solid-state lighting, with the potential for the use of Eu^2+^/Eu^3+^-coactivated calcium silicate nanophosphors to develop white light emission technologies endowed with enhanced color rendering and luminous efficacy.

## 1 Introduction

**Figure 1:**
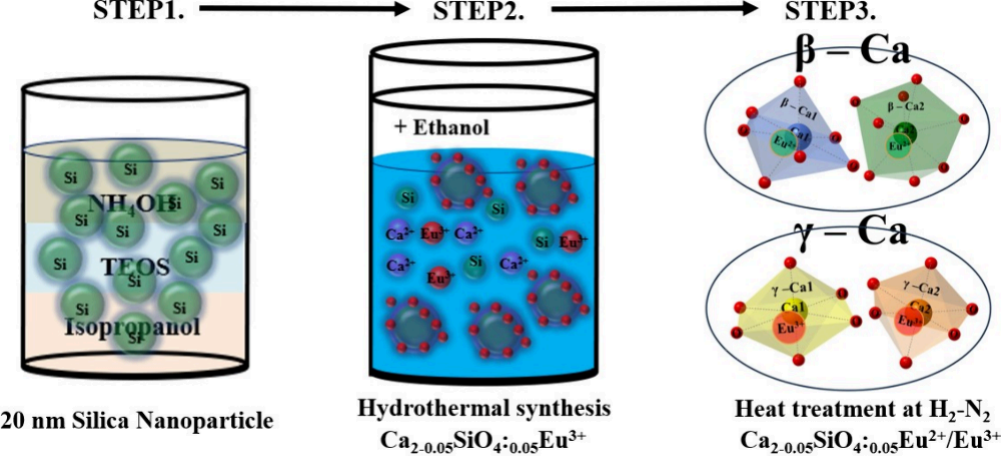
Schematic diagram for the synthesis of trivalent and divalent europium ion-codoped calcium silicate nanoparticles and subsequent reductive heat treatment of the Ca_2–0.05_SiO_4_:_0.05_Eu^2+^/Eu ^3+^ phosphors.

In recent years, there have been significant advancements toward achieving high-quality white light sources in solid-state lighting.^1−3^ The conventional approach for generating white light involves combining a blue InGaN LED with a yellow-emitting phosphor, typically Y_3_Al_5_O_12_:Ce^3+^ (YAG:Ce^3+^).^4−6^ However, this method has inherent limitations, like a notable deficiency in red emission, which results in a low Color Rendering Index (CRI) and a high Correlated Color Temperature (CCT). These limitations render this method unsuitable for critical applications.^7,8^ An alternative approach has emerged aiming to improve the quality of white light emitted by LEDs while achieving a higher CRI. This approach involves employing a UV LED chip emitting 300–410 nm and coating it with three distinct phosphors emitting blue, green, and red light.^9−14^ Despite its promise, this method causes the reabsorption of blue light from red- and green-emitting phosphors and a substantially decreased Stokes shift. This collectively lead to reduced luminous efficiency.^15−17^ Rare-earth ion-containing full-color-emitting phosphors could overcome such challenges, owing to their exceptional luminescent properties, minimal color aberration, and enhanced color rendering. Among these rare-earth ions, europium (Eu) is particularly promising due to the presence of two distinct ionic forms, Eu^2+^ and Eu^3+^, which are essential luminescent species. Eu^2+^ ions typically exhibit intense and broad emissions spanning the blue, blue-green, or green regions, arising from their 4f^6^5d^1^-4f^7^ electronic transitions. In contrast, Eu^3+^ ions emit in the orange-red or red spectral range, originating from their ^5^D_0_-^7^F_*J*_ (*J* = 0–4) transitions.^18,19^ Therefore, phosphors prepared by codoping Eu^2+^ and Eu^3+^ in a single host material can offer effective white light emission because they combine the blue-greenish emission of Eu^2+^ with the red emission of Eu^3+^.^20−25^ In our recent research, we successfully synthesized calcium silicate phosphors doped with trivalent europium ions (Eu^3+^) (Ca_2_SiO_4_:Eu^3+^), demonstrating exceptional luminescence properties, red emission, a high quantum yield (QY), and CCT. This was achieved through successful crystal size control in a hydrothermal synthesis using 20 nm silicate precursor seeds.^26^ In this study, we propose a thermal reduction process to enhance the material’s photoluminescent characteristics through coexistence of Eu^3+^ and Eu^2+^ in Ca_2_SiO_4_. This tuning process had an impact on both the relative ratio of Eu^2+^-to-Eu^3+^ ions and their crystal structure, adjusting the luminescence wavelengths of the phosphors (Figure 1). This work presents opportunities to develop Eu^2+^/Eu^3+^-coactivated single host white light calcium silicate phosphors by strategically manipulating the mixed crystal phases of silicate-based calcium series compounds.

## 2 Materials and Methods

### 2.1 Materials

Tetraethyl orthosilicate (TEOS, 99% Cat# 86578), ammonium hydroxide (NH_4_OH, 28–30%, Cat# A669S-500), europium(III) nitrate hydrate (Eu(NO_3_)_3_·5H_2_O, 99%, Cat# 254061-1G), calcium nitrate tetrahydrate (Ca(NO_3_)_2_·4H_2_O, 99%, Cat# 237124-500G), and polyoxyethylene (5) nonylphenylether, branched (IGEPAL@ CO-520, Cat# 238643-100G) were purchased from Sigma-Aldrich Chemicals (Atlanta, GA).

### 2.2 Synthesis of Silica Nanoparticles (SNP)

Twenty nm silica nanoparticles (SNPs) were synthesized using the *in situ* silica sol–gel method, with some modifications based on previous studies.^26,27^ The procedure began with thoroughly dispersing IGEPAL in cyclohexane through sonication for approximately 10 min and stirring at 30 °C for 30 min to ensure a uniform solution. Next, NH_4_OH and TEOS were added to the reaction mixture while vigorously stirring it overnight at 70 °C, resulting in the formation of spherical-shaped 20 nm SNPs. The size of the SNPs was controlled by adjusting the ratio of NH_4_OH to TEOS to 800 μL:200 μL. Finally, a white precipitate was obtained and washed three times with deionized water.

### 2.3 Synthesis of Coactivated Trivalent and Divalent Europium Ion-Doped Ca_2_SiO_4_

Ca_2_SiO_4_ phosphors coactivated with Eu^2+^/Eu^3+^ ions were synthesized using a hydrothermal reaction with silica nanoparticles. The Ca_2_SiO_4_:Eu^3+^ was prepared through a high-temperature hydrothermal reaction with chemical precursors (or dopants). The silica nanoparticles obtained above were dispersed in ethanol, and an aqueous solution containing a mixture of Ca(NO_3_)_2_·6H_2_O and Eu(NO_3_)_3_·6H_2_O was added. This mixture was initially stirred at 80 °C for 2 h to get a homogeneous solution. The solution then placed in a 20 mL Teflon-lined autoclave, heated at 180 °C for 24 h to obtain the white precipitates, and then sequentially washed with ethanol and deionized water. Thereafter, it was subjected to thermal treatment in atmospheric air at 200 °C for 24 h to decompose the organic compound in the sample. Accordingly, a pure white Ca_2–0.05_SiO_4_:_0.05_Eu^3+^ powder was obtained. The reduction of Eu^3+^ to Eu^2+^ in Ca_2–0.05_SiO_4_:_0.05_Eu^3+^ powder was subsequently annealed at four different temperatures (700, 800, 900, and 1000 °C) for 4 h in a reducing atmosphere of 5% H_2_–95% N_2_. The reduction process and its dependence on the annealing temperature were characterized by various analytical techniques. For comparison, commercial Ca_2–0.05_SiO_4_:_0.05_Eu^3**+**^ was prepared by the solid-state reaction method by finely grinding conventional SiO_2_ (Cat# S5130, Sigma-Aldrich).^31,32^ The obtained products were structurally characterized using powder XRD and TEM. The Ca_2_SiO_4_'s synthesized with a 20 nm silica precursor and subjected to different heat treatment conditions were denoted as C2S_20_-700, C2S_20_-800, C2S_20_-900, and C2S_20_-1000. C2S-C was the notation used for the probe synthesized with commercial silica.

### 2.4 X-Ray Diffraction Studies of Ca_2–0.05_SiO_4_:_0.05_Eu^3+^

The crystalline structure of Ca_2_SiO_4_ was examined using a powder XRD (X’Pert Pro, PANalytical) instrument. The instrument operated under conditions of 40 kV and 40 mA, employing CuKα radiation with a wavelength of 0.154 nm. Measurements were conducted over a 2θ angle range of 10°–80° at a scanning rate of 0.02° per second. The XRD data acquired were meticulously compared and correlated with simulated powder patterns from the Inorganic Crystal Structure Database (ICSD) for a comprehensive analysis and characterization.

### 2.5 Morphological Study of Silica Nanoparticles (SNP) and Ca_2–0.05_SiO_4_:_0.05_Eu^3+^

The particle size and morphology of silica nanoparticles (SNPs) and Ca_2–0.05_SiO_4_:_0.05_Eu^3**+**^ nanoparticles were meticulously evaluated using TEM. The TEM, a JEOL 2200FS model, was operated at an accelerating voltage of 200 kV. This method facilitated detailed observation and measurement of the nanoparticles, enabling precise analysis of their structural attributes.

### 2.6 The PL Studies of Ca_2–0.05_SiO_4_:_0.05_Eu^3+^

The excitation and emission spectra of the Ca_2–0.05_SiO_4_:_0.05_Eu^3**+**^ nanoparticles were obtained by using photoluminescence (PL) spectroscopy (FP-8500, JASCO, Japan) with a 450W Xe lamp as the excitation source. The samples were mounted on a polymer stub, and their excitation and emission were measured by maintaining a constant slit width of 0.1 nm for samples.

### 2.7 XPS

The chemical state was investigated via X-ray photoelectron spectroscopy (XPS), using an AXIS SUPRA+ system (Kratos Analytical Ltd.).

### 2.8 CIE

The Commission International de I’Eclairage (CIE) chromaticity coordinates (*x*, *y*) were calculated from the obtained emission spectra of the samples by using the CIE calculator. The CCT and color purity were also calculated using the obtained diagram of the CIE chromaticity.

## 3 Results and Discussion

### 3.1 Photoluminescence Properties of Prepared Ca_2–0.05_SiO_4_:_0.05_Eu^3+^

**Figure 2:**
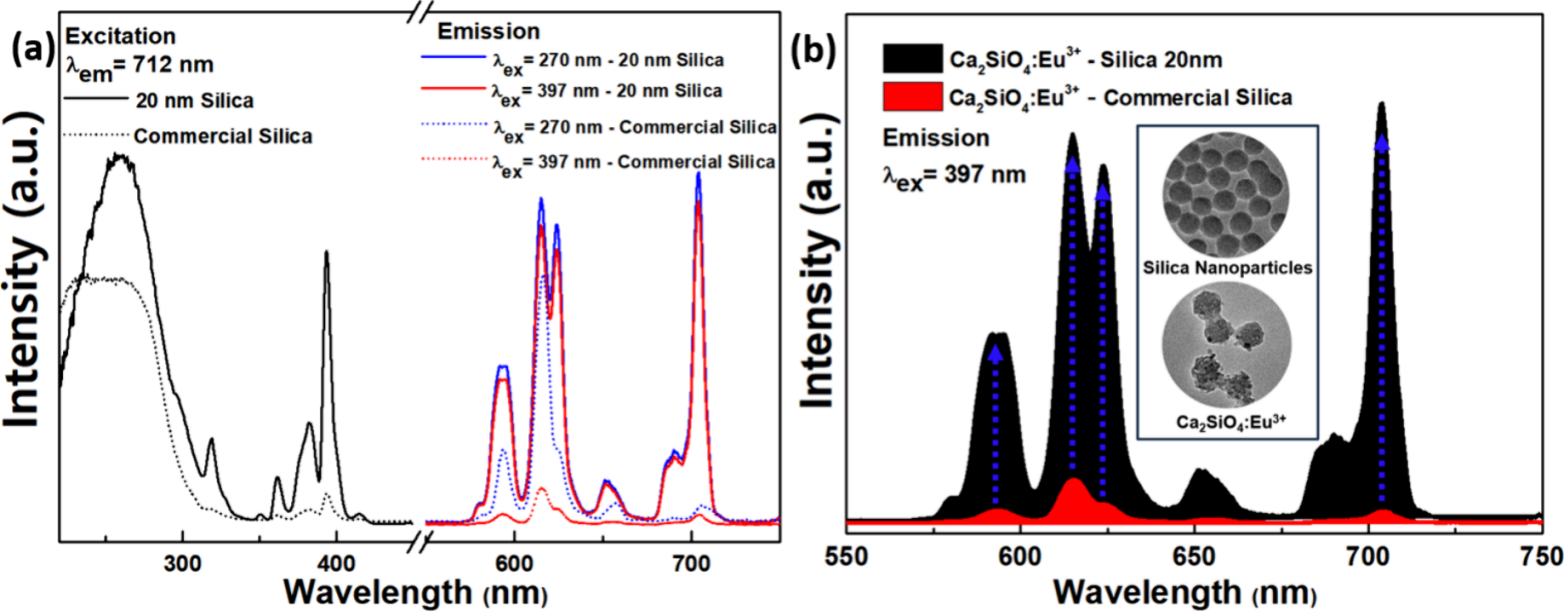
(a) PL excitation at 712 nm (left) and emission spectra excited at 270 and 397 nm (right) of the Ca_2–0.05_SiO_4_:_0.05_Eu^3+^ phosphors prepared with 20 nm silica and commercial silica. (b) Emission spectra and TEM images of Ca_2–0.05_SiO_4_:_0.05_Eu^3+^ phosphors using 20 nm silica and commercial silica.

We synthesized Ca_2–0.05_SiO_4_:_0.05_Eu^3+^ nanoparticles produced via a two-step method with 20 nm silica precursors or commercial silica. Our study analyzed the photoluminescence excitation (PLE) spectra of the phosphors, specifically at 712 nm emission (Figure 2a, left). We observed two distinct peaks appeared at 270 and 397 nm. Compared to the Ca_2–0.05_SiO_4_:_0.05_Eu^3+^ phosphors with commercial silica, the phosphor prepared with 20 nm silica demonstrated a higher excitation efficiency at both wavelengths with even more prominent intensity differences at 397 nm. We next measured the photoluminescence (PL) spectra at 270 and 397 nm excitations (Figure 2a, right). Specifically, under 270 nm excitation, the Ca_2–0.05_SiO_4_:_0.05_Eu^3+^ sample with 20 nm silica showed a 35% increase in emission efficiency across the 575–725 nm spectrum compared to that of the sample with commercial silica. Under 397 nm excitation, the emission efficiency rose 130% in the same spectral range.^26^ These PLE and PL spectra results suggest that the Ca_2–0.05_SiO_4_:_0.05_Eu^3+^ phosphor synthesized with 20 nm silica has superior UV absorption and effectively channels the excitation energy to the Eu^3+^ ions, resulting in efficient PL emission. Conclusively, the Ca_2–0.05_SiO_4_:_0.05_Eu^3+^ nanoparticles prepared with 20 nm silica showed significantly enhanced red emission (quantum efficiency: 87.95%, color purity: 99.8%) and a 400% increase in photoluminescence compared to the commercial silica one at 712 nm transition (Figure 2b). This enhanced optical performance is attributed to structural and spectroscopic alterations due to crystal size control.^26^

### 3.2 Effect of Different Heat Treatment Temperatures on Structure Properties

**Figure 3:**
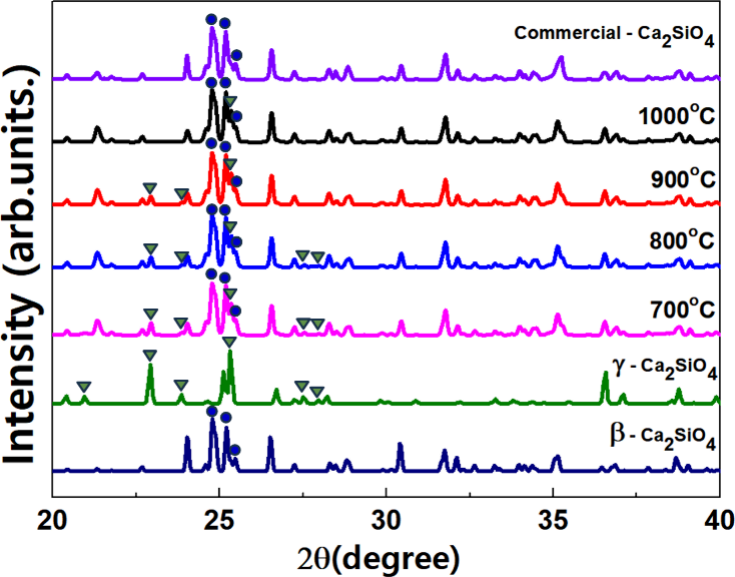
Powder XRD patterns of Ca_1.95_Eu_0.05_SiO_4_ phosphors sintered at various temperatures from 700 to 1000 °C in 5% H_2_–95% N_2_ atmosphere.

We have previously found that the optimal Eu dopant concentration is 5% for Ca_2_SiO_4_:Eu^3+^ nanoparticles.^31,32^ Here, we investigate the effect of the annealing temperature on the crystal structure of the particles. Figure 3 shows the XRD patterns of Ca_1.95_Eu_0.05_SiO_4_ samples annealed in a 5% H_2_–95% N_2_ atmosphere at various temperatures (700, 800, 900, and 1000 °C). The standard database patterns for the γ and β phases are included for comparison in the plot. For the Ca_1.95_Eu_0.05_SiO_4_ phosphor made with 20 nm silicate seeds, the cell parameters of the β type with P121/n1 space are found to be *a* = 5.51, *b* = 6.76, *c* = 9.32 Å, and *V* = 346.1. The space group Pbnm, γ-type Ca_2_SiO_4_ includes the cell parameters of *a* = 5.07, *b* = 11.21, *c* = 6.76 Å, and *V* = 384.7. When heat treated at 700 °C, the resulting Ca_1.95_Eu_0.05_SiO_4_(C2S_20_-700) phosphors consisted of a combination of the stable γ-Ca_2_SiO_4_ (orthorhombic) phase and the metastable β-Ca_2_SiO_4_ (monoclinic) phase. As the temperature increased to 800, 900, and 1000 °C, we observed a reduction in the γ phase and an increase in the β phase. The samples sintered at 1000 °C show a crystal structure of pure β-Ca_2_SiO_4_ phase similar to commercial Ca_1.95_Eu_0.05_SiO_4_(C2S-C). A previous study, by Nakano et al. demonstrated that mixed phases existed as the β- and γ-phases in Ca_2_SiO_4_, with a rise of the β-phase noted at high temperature sintering.^28,29^ Conclusively, sintering at various high temperatures could cause the observable changes in phosphors’ crystal structures, presumably affecting their photoluminescence properties.

### 3.3 Structural and Morphological Analysis of Ca_1.95_Eu_0.05_SiO_4_

**Figure 4:**
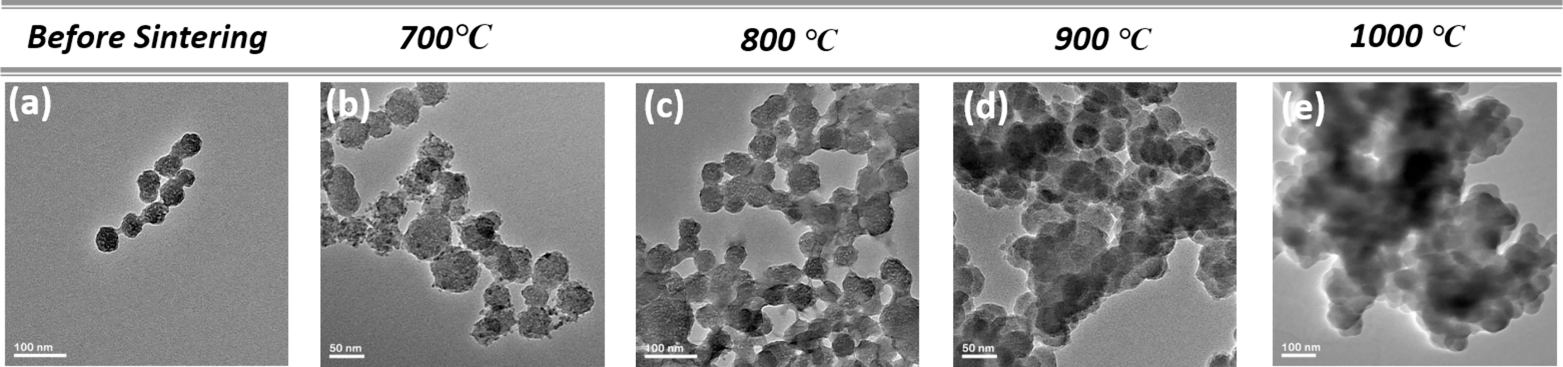
Representative of TEM images of Ca_1.95_Eu_0.05_SiO_4_ phosphors (a) before sintered and (b–e) at various temperatures at 5% H_2_–95% N_2_.

The size and morphological characteristics of the Ca_1.95_Eu_0.05_SiO_4_ phosphors synthesized under various 5% H_2_–95% N_2_ heat treatment temperatures (700, 800, 900, and 1000 °C) are depicted in Figure 4. Specifically, for the Ca_1.95_Eu_0.05_SiO_4_(C2S_20_-700) samples sintered at 700 °C (Figure 4b), a comparison with Figure 4a (before sintering) reveals that the particles manifest a consistent spherical morphology, with diameters falling within the 30–50 nm range. However, as the heat treatment temperature ascends from samples c to e, there is an observable increase in particle size and agglomeration. We speculate that the uniformly distributed, nonagglomerated Ca_1.95_Eu_0.05_SiO_4_ phosphors sintered at 700 °C could cause trivalent europium ions (Eu^3+^) to dominate the emission rather than divalent europium ions (Eu^2+^). Next, we investigated the PL properties of the samples.

### 3.4 Photoluminescence Properties of Prepared Ca_2–0.05_SiO_4_:_0.05_Eu^2+^/Eu^3+^

**Figure 5:**
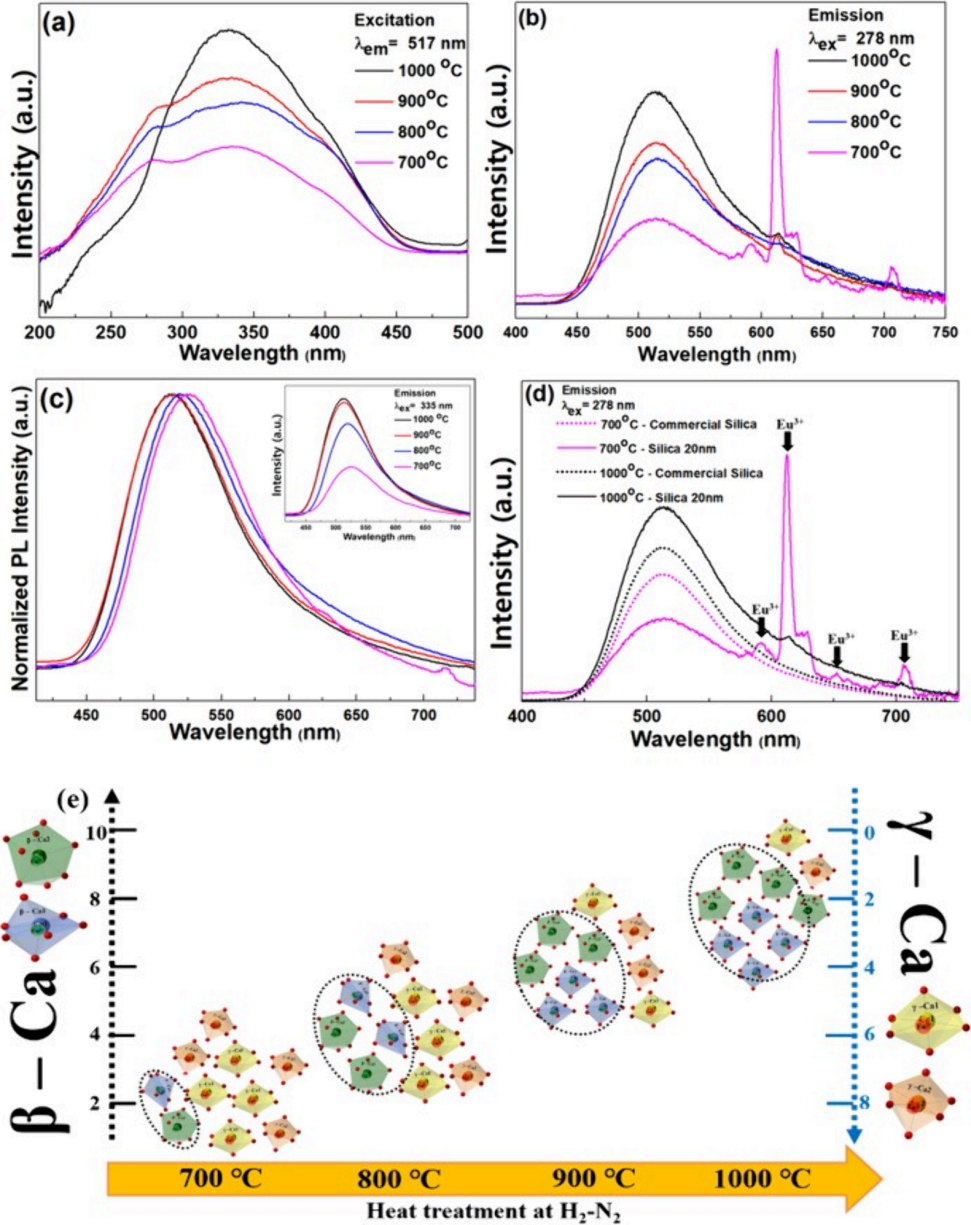
PL excitation at 517 nm (a) and emission spectra excited at 278 nm (b) and 335 nm (c) of the Ca_2–0.05_SiO_4_:_0.05_Eu^2+^/Eu^3+^ phosphors treated with various heat temperatures at H_2_–N_2_. (d) Comparison of PL emission of Ca_2–0.05_SiO_4_:_0.05_Eu^2+^/Eu^3+^ prepared using 20 nm silica precursor to Ca_2–0.05_SiO_4_:_0.05_Eu^2+^/Eu^3+^ using commercial silica. (e) Crystal phase evolution of Ca_2–0.05_SiO_4_:_0.05_Eu^2+^/Eu^3+^ during heat treatment in 5% H_2_–95% N_2._.

The luminescent properties of the Ca_2–0.05_SiO_4_:_0.05_Eu^2+^/Eu^3+^ phosphor are influenced by the relative ratio of Eu^2+^-to-Eu^3+^ ions from the Eu^2+^/Eu^3+^ coactivate phosphors.^19,30^ Xiaoming et al. proposed that the adjustment of active europium ions (Eu^2+^ and Eu^3+^) participates in the PL process either by changing excitation wavelength of UV light or Eu concentration. Wanping et al. concentrated on the synthetic procedure (e.g., reaction time, reducing agent, and Eu concentration) to tune the ratio of Eu^2+^ to Eu^3+^ within the YF_3_:Eu host.^19^ In this study, with a focused variation in the H_2_–N_2_ heat treatment temperature, we modulated the ratio of Eu^2+^ to Eu^3+^ within the Ca_2_SiO_4_ host lattice and examined the PL properties. All other experimental conditions were maintained (e.g., dopant concentration and host composition). Figure 5a,b shows the excitation/emission spectra of the sintered Ca_2–0.05_SiO_4_:_0.05_Eu^2+^/Eu^3+^ phosphor. When the excitation spectra were monitored at 517 nm, which corresponds to the strongest emission intensity, a relatively broad absorption band was revealed spanning the 200–450 nm range. Two distinct peaks appeared, which are attributed to the host–lattice absorption (the first peak at 278 nm) and to the ^4^f_7_–^5^d_1_ transition of the Eu^2+^ ion (the second peak at 335 nm).^31−33^ For all samples with different sintering temperatures, the two primary peaks were consistently observed and were accompanied by a broad wavelength spectrum with an average Full-Width Half Maximum (fwhm) of 175 nm. However, the Ca_2–0.05_SiO_4_:_0.05_Eu^2+^/Eu^3+^ (C2S_20_-1000) phosphor treated at 1000 °C exhibits a smaller fwhm of 145 nm and lacks an excitation peak at 270 nm. The presence of multiple peaks in the Eu^2+^ and Eu^3+^ spectra can be ascribed to variations in excitation energy levels, particularly within the distinct crystal structures of the β and γ phases. Figure 5b illustrates the emission spectrum resulting from irradiation with 278 nm light, which concurrently excites both Eu^2+^ and Eu^3+^ ions as photoactivators. Emission peaks between 580 and 720 nm are observed across all samples. These peaks correspond to 4f → 4f transitions, specifically originating from the ^5^D_0_ excited state to the ^7^F_*J*_ levels (with *J* values ranging from 1 to 6) of the Stark components.^34−36^Figure 5c exhibits a broad emission band in the range of 450–725 nm of the Ca_2–0.05_SiO_4_:_0.05_Eu^2+^/Eu^3+^ phosphors at the excitation wavelength of 335 nm. The emission spectra highlight the variations in the emission wavelength and PL intensity (inset) for the different heat treatment temperatures. As the heat treatment temperature increased, the emission intensity was increased, and the peak position had a slight blue shift. Next, we elucidated the difference in the crystal field environment surrounding the Eu^2+^/Eu^3+^ ions in the samples. We identified that the heat-treated Ca_2–0.05_SiO_4_:_0.05_Eu^2+^/Eu^3+^ samples have mixed phases of β- and γ-Ca_2_SiO_4_. Although both contain two Ca sites, their relevant coordination environments differ. For the β phase, the two Ca sites are 7-fold coordinated Ca(β-1) and 8-fold coordinated Ca(β-2), respectively, and the site symmetry of both sites is C1. Conversely, for the γ-phase, although both Ca sites have an identical coordination number (CN = 6), their site symmetries diverge. Specifically, the Ca(γ-1) site exhibits a Ci symmetry, whereas the Ca(γ-2) site has a Cs symmetry. The emission spectrum exhibits the coexistence of both Eu^3+^ and Eu^2+^ ions demonstrating that not all Eu^3+^ ions are completely reduced to Eu^2+^ during the heat treatment process. The C2S_20_-700 phosphor sample prominently displays Eu^3+^ characteristics with minimal luminous efficiency from Eu^2+^. As the heat treatment temperature increases, the intensity of the Eu^3+^ peak diminishes for the samples, with a simultaneous increase in the Eu^2+^ wavelength peak. From XRD analysis (Figure 3), the Ca_2–0.05_SiO_4_:_0.05_Eu^2+^/Eu^3+^ phosphor was found to have a mixture of β and γ crystal phases, with the proportion of γ crystal phases steadily diminishing with the reductive heat treatment. The sintered samples (C2S_20_-1000) at 1000 °C thus have crystal structures similar to the commercial Ca_1.95_Eu_0.05_SiO_4_(C2S-C) composed of pure β phase (Figure 5e). Interestingly, the emission spectra demonstrate that only the Ca_2–0.05_SiO_4_:_0.05_Eu^2+^/Eu^3+^ samples prepared by 20 nm seed have the conspicuous presence of Eu^3+^ characteristics. No peaks associated with Eu^3+^ ions were detected for the commercial Ca_1.95_Eu_0.05_SiO_4_(C2S-C) and sintered treated Ca_2–0.05_SiO_4_:_0.05_Eu^2+^ samples (Figure 5d). From these findings, we can conclude that the reductive heat treatment can tailor the crystal structures for the Ca_2–0.05_SiO_4_:_0.05_Eu^2+^/Eu^3+^ and the presence of γ phases within their nanostructures contributes to the emissive properties of Eu^3+^ ions.

### 3.5 XPS

**Figure 6:**
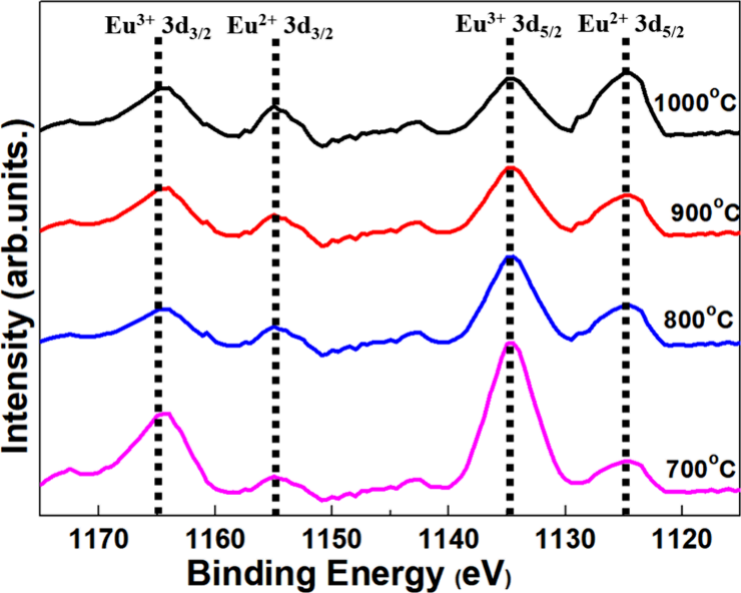
XPS spectra displays binding energy of Eu 3d (Eu^2+^ 3d_5/2_, Eu^3+^ 3d_5/2_, Eu^2+^ 3d_3/2_, and Eu^3+^ 3d_3/2_).

XPS analysis reveals the ratio of Eu^2+^ to Eu^3+^ within the Ca_1.95_Eu_0.05_SiO_4_ samples that were reductively sintered. This approach facilitates a comprehensive understanding of the excitation and emission intensities of the phosphors by adjusting the oxidation status of europium ions (Eu^2+^, Eu^3+^). In the XPS spectra of Eu 3d (Figure 6), distinctive oxidation states of Eu^2+^ and Eu^3+^ are prominently discernible for thermally treated Ca_2–0.05_SiO_4_:_0.05_Eu^2+^/Eu^3+^. Four distinctive components emerged, including Eu^2+^ 3d_5/2_ (1124.9 eV), Eu^3+^ 3d_5/2_ (1133.6 eV), Eu^2+^ 3d_3/2_ (1154.5 eV), and Eu^3+^ 3d_3/2_ (1163.3 eV), and the relative dominance of the Eu^2+^/Eu^3+^ was characterized.^37,38^ The Eu^2+^/Eu^3+^ ratio from the samples was then estimated on the basis of peak analysis. The specific molar ratio (Eu^2+^:Eu^3+^) remains a critical parameter for their photoluminescence.^39−41^ The coexistence ratio of Eu^2+^ and Eu^3+^ was approximately C2S_20_-700 (2.5:7.5), C2S_20_-800 (3.5:6.5), C2S_20_-900 (4.7:5.3), and C2S_20_-1000 (4.2:5.8). Specifically, in the C2S_20_-700 phosphor sample, Eu^3+^ characteristics are prominently displayed (1:3 ratio), with only a minor contribution of Eu^2+^ to the luminescence efficiency. This elucidates that the thermal processing conditions can influence the europium oxidation states (Eu^2+^/Eu^3+^ ratio) and the resulting PL within the phosphors.

### 3.6 CIE and Prototype Warm White LED

**Figure 7:**
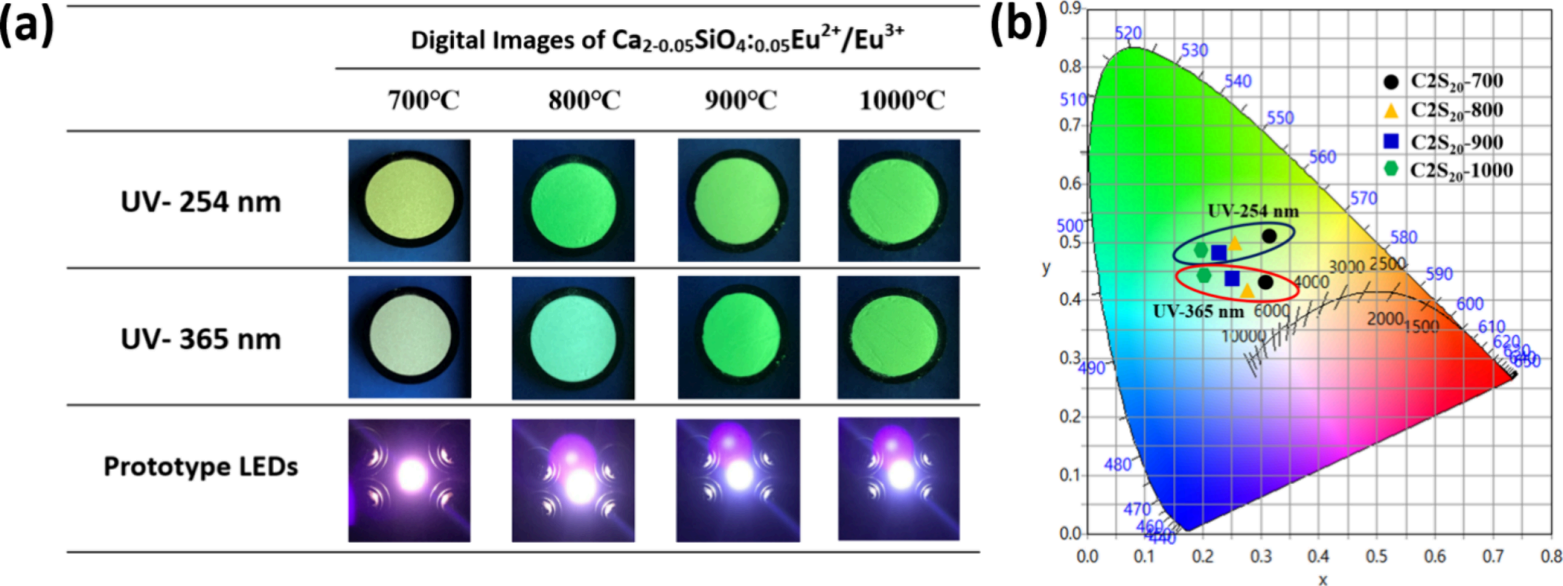
(a) Digital images of Ca_2–0.05_SiO_4_:_0.05_Eu^2+^/Eu^3+^phosphors under UV light at two different wavelengths (254 and 365 nm) and photographs of optimized illuminating prototype LEDs. (b) CIE chromaticity coordinates of phosphors for Ca_2–0.05_SiO_4_:_0.05_Eu^2+^/Eu^3+^.

The color coordinates are critical parameters for evaluating the performance of photoluminescent materials. The CIE chromaticity coordinates were determined from the emission spectra of the samples. As shown in Figure 7a, CIE chromaticity coordinate values of Ca_2–0.05_SiO4:_0.05_Eu^2+^/Eu^3+^ prepared with 20 nm silicate seed with heat treatment can be observed at: C2S_20_-700 = (*x*:0.31, *y*:0.44), C2S_20_-800 = (*x*:0.27, *y*:0.42), C2S_20_-900 = (*x*:0.23, *y*:0.47), and C2S_20_-1000 = (*x*:0.20, *y*:0.48) for 254 nm; C2S_20_-700 = (*x*:0.32, *y*:0.52), C2S_20_-800 = (*x*:0.50, *y*:0.26), C2S_20_-900 = (*x*:0.25, *y*:0.43), and C2S_20_-1000 = (*x*:0.22, *y*:0.47) for 365 nm. A systematic shift in color coordinates was observed, transitioning from a yellow-greenish hue to a greenish-blue hue in response to an increase in the 5% H_2_–95% N2 reduction temperature. This result elucidates the nuanced interplay of thermal treatment and oxidation states in tuning the chromatic properties of the synthesized phosphors to enhance the materials adaptability and performance in varied photoluminescent applications. To evaluate the color simulations, the prototype LEDs have been fabricated by impregnating the prepared Ca_2–0.05_SiO4:_0.05_Eu^2+^/Eu^3+^ phosphors in 335 nm LED chips. Figure 7b presents a fabricated prototype white-light-emitting diode (w-LED) that demonstrates a versatile warm-to-cold white light. Conclusively, the diverse white light manifestation could be realized through the controlled oxidation ratio of Eu^3+^ and Eu^2+^ of the sintered Ca_2–0.05_SiO_4_:_0.05_Eu^2+^/Eu^3+^ nanophosphors. There is no need to change the type of rare-earth ion within a single host material. This investigation could lead to substantial adaptability and applicability across diverse lighting environments, which demand varied color temperatures.

## 4 Conclusions

This study elucidates the influential role of nanosilicate size and thermal treatment conditions on manipulating the photoluminescent properties of Ca_2–0.05_SiO_4_:_0.05_Eu^2+^/Eu^3+^ nanophosphors. Our exploration revealed that different sintering temperatures impart significant variations in the crystallographic structures of the samples. An incremental increase in treatment temperature resulted in a discernible enlargement of particle size and aggregation. We observed an evident modulation in the emission characteristics of the phosphors attributed to the variance in the oxidation states of europium ions (Eu^2+^ and Eu^3+^). The thermally controlled reduction process had a substantial effect on the Eu^2+^-to-Eu^3+^ ion ratio, particle size, and overall crystal structure of the phosphors, thus dictating their luminescent properties. XPS validated the precise control of the europium ion oxidation states, affirming the robust influence of the reduction process on tailoring material composition. Furthermore, the prototype LEDs fabricated from the synthesized phosphors demonstrated a wide adaptability in emitting versatile white light from warm to cold, a significant advancement in photoluminescent applications. In conclusion, this research provides invaluable insights into manipulating synthesis parameters, such as nanosilicate sizes and thermal treatment, to optimize the photoluminescent properties of Ca_2–0.05_SiO_4_:_0.05_Eu^2+^/Eu^3+^ phosphors. The findings herald promising prospects in enhancing efficacy and applying these materials in diverse luminescent technologies, notably in the fabrication of high-efficiency white LEDs.
